# Exploring the use of music to promote physical activity: From the viewpoint of psychological hedonism

**DOI:** 10.3389/fpsyg.2023.1021825

**Published:** 2023-01-25

**Authors:** Kyoung Shin Park, David M. Williams, Jennifer L. Etnier

**Affiliations:** ^1^Department of Kinesiology, University of North Carolina at Greensboro, Greensboro, NC, United States; ^2^Center for Health Promotion and Health Equity, Brown University, Providence, RI, United States

**Keywords:** affect, exercise, entrainment, motivation, reward, rhythm, pleasure, arousal

## Abstract

Despite the global efforts to encourage people to regularly participate in physical activity (PA) at moderate-to-vigorous intensity, an inadequate number of adults and adolescents worldwide meet the recommended dose of PA. A major challenge to promoting PA is that sedentary or low-active people experience negative shifts in affective valence (feeling bad versus good) in response to moderate-to-vigorous intensity PA. Interestingly, empirical data indicate that listening to music during acute bouts of PA positively alters affective valence (feeling good versus bad), reduces perceived exertion, and improves physical performance and oxygen utilization efficiency. From the viewpoint of the ancient principle of psychological hedonism – humans have ultimate desires to obtain pleasure and avoid displeasure – we elaborate on three putative mechanisms underlying the affective and ergogenic effects of music on acute bouts of PA: (1) musical pleasure and reward, (2) rhythmic entrainment, and (3) sensory distraction from physical exertion. Given that a positive shift in affective valence during an acute bout of PA is associated with more PA in the future, an important question arises as to whether the affective effect of music on acute PA can be carried over to promote long-term PA. Although this research question seems intuitive, to our knowledge, it has been scarcely investigated. We propose a theoretical model of Music as an Affective Stimulant to Physical Activity (MASPA) to further explain the putative mechanisms underlying the use of music to promote long-term PA. We believe there have been important gaps in music-based interventions in terms of the rationale supporting various components of the intervention and the efficacy of these interventions to promote long-term PA. Our specification of relevant mechanisms and proposal of a new theoretical model may advance our understanding of the optimal use of music as an affective, ergogenic, and sensory stimulant for PA promotion. Future directions are suggested to address the gaps in the literature.

## 1. Introduction

Mounting evidence indicates that regular physical activity (PA) is associated with numerous health benefits (Warburton et al., 2006; Reiner et al., 2013) whereas insufficient PA is found to be the fourth leading cause of global mortality, accounting for approximately 3.2–5 million deaths every year (Lee et al., 2012). As such, serious efforts have been made at a global and national level to encourage people to regularly engage in PA at work, in transit, or for leisure (Haskell et al., 2007; Piercy et al., 2018; Bull et al., 2020). Public health officials recommend moderate-to-vigorous intensity PA (MVPA) for 150–300 min/week for adults and for at least 60 min/day for children and adolescents (Bull et al., 2020). However, it has been reported that approximately 30% of adults and 80% of adolescents worldwide do not reach the recommended levels of MVPA (WHO, 2020), and the rates further decline in obese and elderly populations (Bennie et al., 2019; Du et al., 2019; Lee et al., 2020), especially in high-income countries (Hallal et al., 2012; Piercy et al., 2018). Overweight and obese adults’ MVPA rate is 20–50% lower than healthy weight adults (Bennie et al., 2019), and a more progressive decline in PA is seen after ages 60–65 (Troiano et al., 2008; Du et al., 2019) when the health benefits of PA become pronounced. Despite the global and national efforts to communicate the health benefits of PA, the rate of PA is lower than desired, which can bring about harmful sequelae for society and individuals (Kohl et al., 2012).

The psychosocial factors predicting or causing PA behaviors have attracted scientific interest (for reviews, see Marcus et al., 1996; Brand and Cheval, 2019; Biddle et al., 2021; Huffman et al., 2021; Stults-Kolehmainen et al., 2022). Over the past two decades, the motivational role of “*affect*,” a gestalt construct with valence (pleasure versus displeasure) and arousal (high versus low) dimensions (Russell, 1980), has received increasing attention as a key factor eliciting motivation for PA (Ekkekakis and Petruzzello, 1999; Ekkekakis, 2003, 2017; Williams, 2008; Rhodes et al., 2009; Rhodes and Kates, 2015; Lee et al., 2016; Murphy and Eaves, 2016; Williams and Bohlen, 2019; Stevens et al., 2020; Stults-Kolehmainen et al., 2020). This is an important direction because a major challenge to promoting PA is that low-active people often experience more negative affective valence than high-active people in response to MVPA (Ekkekakis et al., 2011). This negative affective response is a critical barrier to adoption and maintenance of PA because, according to the ancient principle of psychological hedonism, human behaviors are driven by a propensity to maximize pleasure and minimize displeasure (Cabanac, 1992; Kahneman et al., 1997; Williams, 2018; Williams, 2019; Young, 1952). This means that when people *like* – or at least *do not dislike* – a single session of PA (hereafter referred to as acute PA), they are more likely to repeat it on a regular basis. This notion is supported by a systematic review showing that a positive shift in affective valence during an acute exercise bout predicted PA in the future, whereas negative shifts in affective valence predicted less PA (Rhodes and Kates, 2015). An affect-based approach to PA promotion may be a timely and important strategy (Ekkekakis, 2017; Williams and Bohlen, 2019; Stevens et al., 2020; Stults-Kolehmainen et al., 2020).

Scientists have demonstrated that music can have motivational effects on PA (Karageorghis and Terry, 1997; Karageorghis et al., 1999; Priest et al., 2004; Karageorghis and Priest, 2012a; Bigliassi et al., 2016). A recent meta-analysis of 139 studies revealed that music listening prior to or during acute PA increases positive affective valence (*g* = 0.48, CI [0.39, 0.56]), reduces ratings of perceived exertion (RPE; *g* = 0.22, CI [0.14, 0.30]), enhances physical performance (*g* = 0.31, CI [0.25, 0.36]), and improves oxygen utilization efficiency (VO2max; *g* = 0.15, CI [0.02, 0.27]) compared to PA without music (Terry et al., 2020). Moderation meta-analyses further revealed that the beneficial effects of music on affective valence and RPE are present across the full range of PA intensity (Terry et al., 2020). These findings substantiate the idea that music makes PA more joyous, less arduous, and more energetic and efficient. In other words, music becomes an affective and ergogenic stimulant to PA. This raises a research question about its underlying mechanism: How does music provide such effects on PA? Although conceptual models have been proposed to address this research question (Karageorghis et al., 1999; Karageorghis, 2015; Clark et al., 2016a), these models do not incorporate concurrent theories of motivation and thus lack a thorough theoretical foundation. Hence, in Section 2, we propose a theoretical model to elucidate the putative mechanism underlying the effects of music stimulation for acute PA from the views of psychological hedonism. In our description of the mechanisms, we suggest methodologies of music manipulation (i.e., beat-accentuation, tempo-synchronization, and personally preferred music selection) that may maximize the effects of putative mechanisms.

The evidence that music Increases positive affective valence and reduces RPE during acute bouts of PA implies that music leads to more positive evaluations of PA sessions – to like it more or dislike it less. This raises another research question: Can the affective effects of music on PA be carried over from acute to long-term phases to motivate regular PA? Although the answer seems intuitive, to our knowledge, this research question has been scarcely investigated and a theoretical basis has not been proposed. However, there is some empirical evidence supporting this possibility. A randomized control trial (RCT) revealed promising results that walking-for-exercise with personally-preferred, beat-accented, tempo-synchronous music playlists substantially increased the weekly volume of PA over 3 months compared with the same exercise prescription with non-beat-accented music playlists or without music among midlife-to-older adults in a home-based cardiac rehab program (Alter et al., 2015). We discuss these findings in comparison with other evidence in the literature (Section 3) and elaborate on the long-term implications of the theoretical model to further our understanding of the potential effects of music for increasing and maintaining regular PA – hereafter referred to as long-term PA (Section 4). Given that prior conceptual models of music stimulation are limited to acute PA (Karageorghis et al., 1999; Karageorghis, 2015; Clark et al., 2016a), our theoretical approach is the first of its kind for the specification of putative mechanisms underlying the use of music stimulation for the promotion of long-term PA.

Music-based interventions have been widely developed and implemented in varying fields of science and medicine (Chen et al., 2022) to treat neurologic (for reviews, see Thaut, 2005; Schaefer, 2014; Leggieri et al., 2019), cardiovascular (for a review, see Chair et al., 2021), or psychiatric conditions (for reviews, see Zhao et al., 2016; Aalbers et al., 2017). In particular, music has been frequently employed to activate the motor system as part of rhythmic auditory stimulation (RAS). RAS refers to an application of pulsed rhythmic auditory stimuli (e.g., metronome and/or music) for the facilitation of body movements that are intrinsically rhythmic (e.g., walking; Thaut et al., 2016). Frequently utilized in clinical settings, people with neurodegenerative diseases – most frequently Parkinson’s disease (PD) and stroke – or brain injuries have benefited from being trained to synchronize their walking steps to RAS (Thaut, 2005; Thaut et al., 2015). RAS interventions have used varying forms of synchronous stimuli - auditory tempo (beats/min, BPM) matched to individual cadence (steps/min) - such as metronome pulse (see reviews by Lim et al., 2005; Nombela et al., 2013; Ghai et al., 2018), contemporary music (de Bruin et al., 2010; Park et al., 2020, 2021), or contemporary music with sonically-enhanced (accentuated) beats (Thaut et al., 1996; McIntosh et al., 1997; Benoit et al., 2014). The evidence that RAS facilitates rhythmic motor behaviors implies its beneficial application for PA promotion because PA is defined as body movement of skeletal muscles that leads to energy expenditure (Caspersen et al., 1985; Ainsworth et al., 2000). However, this idea has not been systematically investigated or theoretically discussed. Given the growing demand for PA promotion in the realm of public health, it is important to delve into the literature to inform future music-based interventions for PA promotion.

## 2. Putative mechanisms of music stimulation for acute physical activity

*The Theory of Hedonic Motivation* (THM) – a theory of psychological hedonism formulated by Williams (2018, 2019) – serves as a theoretical basis for our approach herein. According to the THM, ‘liking’ and ‘disliking’ (i.e., Berridge, 2003) represent the neurobiological underpinnings of *hedonic responses* – an organism’s *automatic* and *immediate* experience of pleasure versus displeasure (i.e., affective valence) in response to a behavior or immediate behavioral outcome. Moreover, ‘wanting’ and ‘dread’ (i.e., Berridge, 2003) represent the neurobiological underpinnings of *hedonic motivation* – a neurobiological process that is automatically triggered by a stimulus and manifests as a felt *hedonic desire* to produce an immediate behavioral outcome that has previously brought immediate pleasure (or relief from displeasure) or a felt *hedonic dread* of producing an immediate behavioral outcome that has previously brought immediate displeasure (or reduced pleasure).

Hedonic motivation is also considered to be an *affectively-charged motivation state* (ACMS), first introduced by Kavanagh et al. (2005), for the conceptualization of *desires*, *wants*, *cravings*, and *urges* for appetitive behaviors (e.g., smoking, drinking, eating). Consistent with the THM, and applied to PA behaviors, Stults-Kolehmainen et al. (2020) conceptualized *urges*, *wants*, *desires*, *dread*, *craving*, and *aversion* as “ACMS and associated feelings that signal a pressing need to approach or avoid a state of muscular movement (or, conversely a state of rest)” (p. 2). Stults-Kolehmainen et al. (2020) further proposed the WANT model (Wants and Aversions for Neuromuscular Tasks), a descriptive, circumplex model of ACMS to move and rest on a continuum of approach and avoidance orientation. In accordance with neurobiological evidence (Desmurget and Sirigu, 2012), the WANT model enunciates that urges, cravings, and dread to move/rest are more intense motivational states than desires, wants, and aversions to move/rest. We adopt this conceptualization in our theoretical approach proposed herein.

**Figure 1:**
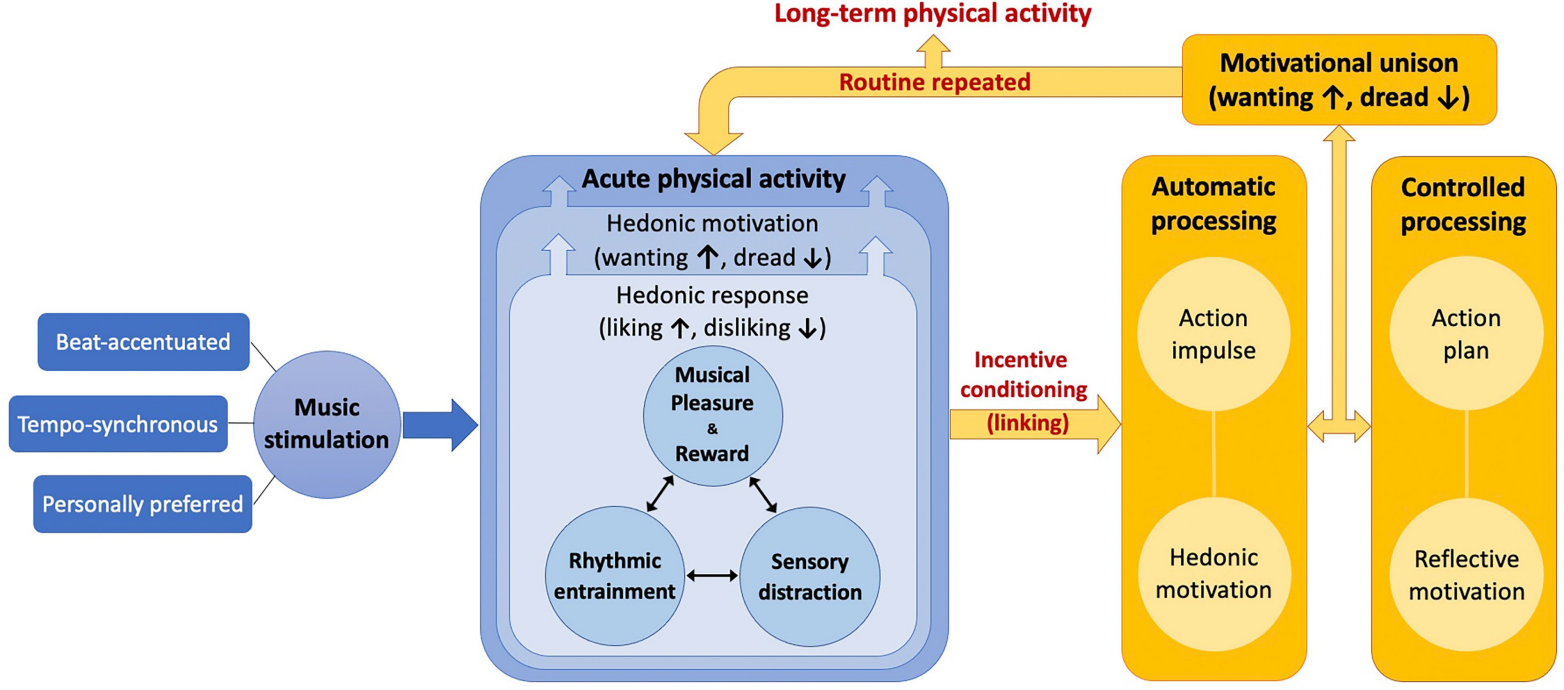
A theoretical model named “Music as an Affective Stimulant for Physical Activity” (MASPA). Acute phase of physical activity is depicted in blue whereas long-term phase of physical activity is illustrated in yellow. Regularly repeating acute routine can lead to long-term physical activity.

Our approach is that hedonic response and hedonic motivation are the core psychological mechanisms underlying affective, ergogenic, and motivational effects of music on PA. Adding music to PA evokes hedonic responses (e.g., positive affect, reduced RPE, exercise enjoyment, arousal, etc.) and this leads to hedonic motivation or ACMS for continuing movement during acute bouts of PA. We propose a theoretical model named “Music as an Affective Stimulant for Physical Activity” (MASPA) to explain this process (see Figure 1). The model contains the following components as the putative psychological mechanisms in acute bouts of PA: (1) musical pleasure and reward, (2) rhythmic entrainment, and (3) sensory distraction from physical exertion. In the following sections, we elaborate on each element of the mechanisms based on relevant theories and empirical evidence from behavioral and neurobiological studies.

### 2.1. Musical pleasure and reward

Many people take pleasure in listening to music. Neurobiological studies have demonstrated that the reward system in the brain is the core of this experience. In a non-musical context, as explained by the *incentive salient hypothesis* (Berridge and Robinson, 1998, 2016; Berridge, 1999, 2007), the cardinal reward stimuli in humans such as money, sex, or food convey feelings of pleasure (liking) and thus motivate the individual to seek the same stimuli again (wanting), although “wanting” can occur without “liking” on some occasions (Berridge, 2007; Berridge and Robinson, 2016). A meta-analysis of 87 neuroimaging studies confirmed the role of the mesolimbic dopaminergic system in processing these fundamental rewards in humans (Sescousse et al., 2013). Interestingly, the mesolimbic dopaminergic system in the human brain is activated by music listening especially when music stimuli are perceived as intensely pleasurable (Zatorre and Salimpoor, 2013; Koelsch, 2014; Zatorre, 2015). This means that – like other reward stimuli in humans – music listening evokes positive hedonic experiences (liking) and this leads to hedonic motivation (wanting) for keeping up with the hedonic experiences. The term, *musical pleasure*, has been derived from these findings to represent music-evoked pleasure responses (Gebauer et al., 2012; Zatorre, 2015; Goupil and Aucouturier, 2019).

Musical pleasure is often accompanied by an arousal response that is defined as physiological activation of the autonomic nervous system (Frijda, 2009) and is another core dimension of the circumplex model of affect (Russell, 1980). One powerful experience of highly-aroused musical pleasure is “thrills.” A *thrill* is “a subtle nervous tremor caused by intense emotion or excitement (as pleasure, fear, etc.), producing a slight shudder or tingling through the body; a penetrating influx of feeling or emotion” (Oxford English Dictionary, 2022). Questionnaire data have shown that this phenomenon is commonly experienced with physical sensations such as shivers or goose pimples by multiple groups of music listeners (Goldstein, 1980; Sloboda, 1991; Panksepp, 1995). The term, *chills*, rather than thrills, was suggested by Panksepp (1995) for its more common use by college-aged adults, and this nomenclature has been followed by successive researchers (Rickard, 2004; Grewe, 2005; Grewe et al., 2007, 2009; Salimpoor et al., 2009). Musical chills have been found to be composed of subjectively reported musical pleasure (Salimpoor et al., 2009) as well as objectively measured changes in physiological arousal such as increased heart rate (HR), respiration, electrodermal activity, and muscle tension as well as decreased body temperature (Blood and Zatorre, 2001; Grewe, 2005; Grewe et al., 2007, 2009; Salimpoor et al., 2009, 2011).

Zatorre and colleagues have demonstrated neurochemical correlates of musical pleasure and chills. The first neuroimaging study with positron emission tomography (PET) – a technique showing the chemical and functional changes in the brain – reported evidence of neural responses to intense musical pleasure by comparing brain activities while listening to self-chosen pleasurable music compared to other-chosen neutral music (Blood and Zatorre, 2001). Results showed that subjective ratings of chill intensity were associated with changes in HR, muscle tension, and respiration depth as well as cerebral blood flow changes in the mesolimbic dopaminergic circuits. They conducted a subsequent PET study with [^11^-C]raclopride – a technique measuring dopamine release in cerebral tissues – and revealed endogenous dopamine release in the reward circuits when healthy adults listened to self-chosen pleasurable music versus other-chosen neutral music, and the magnitude of the mesolimbic dopamine activity was associated with the frequency and intensity of chills and the degree of pleasure (Salimpoor et al., 2011). More recently, researchers conducted a double-blind pharmacological trial with oral administration of a dopamine precursor (levodopa) that caused greater responses in musical pleasure, chills, and reward compared with a dopamine antagonist (risperidone) and a placebo (lactose; Ferreri et al., 2019). These findings show that people like to listen to music (hedonic responses) and thus they seek more music-related activities (hedonic motivation) and dopamine modulates this process.

The findings we described above have been accrued in non-PA contexts. In PA settings, Terry et al.’s (2020) meta-analysis confirms that listening to music prior to or during acute PA evokes more positive hedonic responses than non-music PA. This finding derived from 29 studies assessing core affective valence during acute bouts of PA in laboratory settings. Arousal responses to music can also occur in PA settings. Although empirical studies are scarce, Karageorghis and colleagues have provided some evidence to uphold this perspective. When high-active adults completed 6-min of vigorous-intensity cycle ergometry under different conditions, subjective ratings of arousal were higher when exercising with synchronous music compared with a synchronous metronome and a no-music control (Lim et al., 2014). When male athletes listened to different music stimuli before performing a hand-grip dynamometer test, fast-tempo/loud music increased perceived arousal and affective valence and improved grip strength performance more than fast-tempo/quiet, slow-tempo/loud, slow-tempo/quiet, and no-music control conditions (Karageorghis et al., 2018). Music can evoke physiological arousal without changes in affective valence, yet whether this can lead to hedonic motivation is uncertain. For example, fast, loud music typically has increased sound pressure levels that intensify arousal regardless of how the music is hedonically appraised (Van Dyck, 2019). From the viewpoint of THM, music-evoked arousal can contribute to hedonic motivation for PA only if it is combined with feelings of pleasure. For example, if certain music evokes high arousal along with positive valence (e.g., eager anticipation, excitement), it can lead to a desire for continuing or repeating the behavior associated with such music. On the other hand, if certain music evokes high arousal combined with negative valence (e.g., anxiety, nervousness), it may lead to a dread of the behavior associated with such music.

As depicted in Figure 1, we propose that musical pleasure serves as the core of hedonic responses to music in PA contexts – similar to non-PA contexts – and this activates the reward system in the brain eliciting hedonic motivation for continuing PA. Future studies may test this mechanism by assessing important markers of musical pleasure, physiological arousal, self-perceived subjective arousal, and/or the frequency and intensity of chills in response to music stimulation during acute PA. This motivational enhancement could lead to the ergogenic effects of music on PA, evidenced by improved physical performance in the meta-analysis (Terry et al., 2020). In other words, music listening makes a PA session more enjoyable and thus enables the PA participant to work more or harder. Because music preference varies by individual factors such as age (Priest et al., 2004), we suggest the use of personally-preferred music playlists that would elicit more hedonic and arousal responses and hedonic motivation for music-based PA, in agreement with views of exercise scientists (Karageorghis and Priest, 2012a,b; Clark et al., 2016a,b; Hutchinson et al., 2018) and neuroscientists (Blood and Zatorre, 2001; Salimpoor et al., 2009, 2011).

### 2.2. Rhythmic entrainment

Music is composed of ordered sounds (*notes*) and silences that recur in time. To untangle the architectonics of music, we here define the key elements of the temporal organization of music – *pulse*, *beat*, *meter*, and *rhythm*. A *pulse* is one of a series of periodic, precisely equivalent stimuli (e.g., metronome ticks) that delineate equal units in the temporal continuum (Cooper and Meyer, 1960). Pulses are referred to as *beats* when being counted within a metric context. *Meter* refers to “the measurement of the number of pulses between regularly recurring accents – marked for consciousness – relative to others” (Cooper and Meyer, 1960, p. 4), which indicates that meter exists only when music listeners perceive beats accented relative to others (Large and Kolen, 1994). *Rhythm* is traditionally defined as “the way in which one or more unaccented beats are grouped in relation to an accented one” (Cooper and Meyer, 1960, p. 6) or more simply, “the serial pattern of variable note durations in a melody.” (Schulkind, 1999, p. 896) Although this definition counts rhythm as an objective property of music, it can also be subjective in that rhythmic experience occurs based on the intricate interactions between other components of music (e.g., pitch, tempo, harmony, timbre), individual differences in capacity and perception, and environments. From a psychological viewpoint, rhythm represents the patterns of durational proportions that are phenomenally present in music, whereas meter involves the initial perception and ensuing anticipation of a series of beats that are extracted from the rhythmic structure of music as it unfolds in time (London, 2012).

London (2012) defines meter as a “musically-specific form of *entrainment*, the synchronization of attention and/or other behaviors (especially motor behaviors) with periodic rhythms in the environment.” (p. 10) Entrainment allows the listener to focus attention on the salient temporal events in music which is important because attention is selective by its nature. It should be noted that the periodicity of meter enables anticipation that fosters entrainment. The motivational effects of music on motor behaviors in humans have been explained in extensive literature reviews, which all point to entrainment as the key principle – from evolutionary and psychological perspectives (Levitin et al., 2018) and from a multi-disciplinary approach combining the principles of physics and neurobiological modeling of brain and oscillatory activities (Damm et al., 2020). Entrainment to musical rhythm has been demonstrated in various motor behaviors including bouncing and clapping (Tranchant et al., 2016), walking (Wittwer et al., 2013; Franěk et al., 2014; Metcalfe, 2016), running (Simpson and Karageorghis, 2006; Terry et al., 2012; Bood et al., 2013; Van Dyck et al., 2015), and free dancing (Burger et al., 2013). Rhythmic motor entrainment to music is a behavior primarily observed in humans but also evident in some animals (Wilson and Cook, 2016). In humans, this psychophysical phenomenon universally appears across cultures, regions, and eras (Cross, 2001) in people of all ages from infants (Zentner and Eerola, 2010) to children (Provasi and Bobin-Bègue, 2003) and young to older adults (Thaut et al., 1992; Metcalfe, 2016; Rose et al., 2021) and in individuals with neurologic impairments (Thaut et al., 2015).

Neuroscientists have demonstrated neural representation of rhythmic entrainment in the brain. According to Large and colleagues’ neural resonance theory (NRT), nonlinear coupling of two oscillatory neutral networks – the auditory system receiving the physical properties of music stimulus and the motor system that integrates sensory inputs from the auditory system – leads to a common oscillatory activity at the beat frequency of music, and such entrained oscillations lead to the formation of rhythm perception (Large and Snyder, 2009). The research team tested the NRT through a series of experiments with electroencephalography (EEG) and magnetoencephalograms (MEG) recordings (Snyder and Large, 2005; Large et al., 2015; Tal et al., 2017). They demonstrated that neural activity increased in the auditory cortex at the same frequency as auditory beats and they determined that such neural oscillation is entrained to the beats because increased neural activity was found even at times when there were missing beats, and the strength of neural responses was correlated with individuals’ speed in perceiving the pulse. According to *the action simulation for auditory prediction hypothesis*, stimulation of rhythmic movement in the motor planning regions of the brain provides neural signals to the auditory system where anticipation of upcoming beats is made (Patel and Iversen, 2014). In support of this notion, brain studies with functional magnetic resonance imaging (fMRI) have observed bilateral activation of the motor regions in the brain during beat perception tasks without overt motor actions (Grahn and Brett, 2007; Chen et al., 2008).

Rhythmic entrainment may underlie the effects of music on exercise performance. Terry et al. (2020) revealed that (a) *tempo-synchronous music* yielded marginally more beneficial effects on performance of acute PA (*g* = 0.44, CI [0.22, 0.65]) than *tempo-asynchronous music* (*g* = 0.31, CI [0.24, 0.38]) and that (b) fast-tempo music (*g* = 0.38, CI [0.30, 0.45]) led to more beneficial effects on exercise performance than slow/medium tempo music (*g* = 0.21, CI [0.14, 0.27]). These findings imply the ergogenic effects of rhythmic entrainment. However, Terry et al. (2020) noted that studies using tempo-synchronous music in PA contexts are relatively rare compared to tempo-asynchronous music, and thus the specific role of entrainment in enhancing exercise performance or promoting long-term PA has not been fully tested. Future studies need to address this gap in the literature. Although we discussed its role in altering physical performance, rhythmic motor entrainment to music can be also accompanied by affective responses. We discuss this idea in the following section.

### 2.3. Rhythmic entrainment as an affective response to music

Rhythmic entrainment typically appears in motor behaviors and is also considered to be a part of the psychological mechanisms underlying affective or emotional responses to music (Juslin and Västfjäll, 2008; Trost and Vuilleumier, 2013; Juslin et al., 2014; Trost et al., 2017). A classic example of coupling motor and affective responses to music is seen from people moving along with pleasant feelings when listening to their favorite songs. Zentner and Eerola (2010) provided critical behavioral evidence that preverbal infants demonstrated increased rhythmic movements when hearing musical or rhythmic stimuli (e.g., Mozart, Saint-Saëns, children’s song, or drumbeats) compared with non-musical stimuli (e.g., adult speech). Interestingly, it was further reported that (a) infants’ rhythmic motions coincided with positive affect, evidenced by a noticeable correlation between the duration of movement and the duration of smiles (*r* = 0.30 and 0.37 in two experiments) and that (b) infants’ duration of smiles showed meaningful correlations with their degree of music-movement synchronization accuracy (*r* = 0.42 and 0.26 in two experiments). These findings indicate that humans are predisposed to rhythmically engage in music with a concurrent display of positive affective states. Zentner and Eerola (2010) noted that “this association raises the possibility that surges in positive affect may facilitate, and perhaps even motivate, rhythmic engagement with metrically regular sound patterns.” (p. 5771).

The co-occurrence of affective and motor responses to music has been further explained by a sensorimotor phenomenon called *groove*. Janata et al. (2012) defined groove as “[an] aspect of the music that induces a pleasant sense of wanting to move along with music.” (p. 54) and also the feeling of being in the groove as having “the urge to move in response to music, combined with the positive affect associated with the coupling of sensory and motor processes while engaging with music (referred to as sensorimotor coupling) in a seemingly effortless way” (p. 56). Groove is considered to be a subjectively-felt sensation and also a type of ACMS for the initiation and maintenance of body movements in response to music (Stults-Kolehmainen et al., 2020). According to the WANT model (Stults-Kolehmainen et al., 2020), this urge to move with music is considered to be a stronger motivational state than a simple desire or want to move. Janata et al. (2012) described a pleasurable drive toward motor actions as the key concept of groove based on the findings that high-groove music stimuli were perceived as more enjoyable and induced a greater amount of spontaneous movements across all body parts (most notably head and feet) during a hand-tapping task, compared with low-and moderate-groove music. Janata et al. (2012) further found that faster-tempo music (115.6 ± 8 BPM) is more groove-inducing than slower-tempo music (90.8 ± 6.6 BPM) and that groove ratings are closely associated with ratings of enjoyment (*r* = 0.82) and familiarity (*r* = 0.57). In an online survey, young-to-midlife adults reported the degree of wanting to move and the feelings of pleasure they experienced while listening to a series of drumbeats in varying ranges of rhythmic patterns (Witek et al., 2014). The results showed a strong correlation between the ratings of wanting to move and the experience of pleasure (*r* = 0.964).

Neuroimaging evidence supports that rhythmic motor entrainment to music occurs as part of affective responses in the brain. In an fMRI study, Kornysheva et al. (2010) showed that listening to preferred musical rhythm that was rated as more beautiful and pleasant resulted in increased activation of motor-related brain regions such as the premotor cortex and cerebellum compared with non-preferred musical rhythm. In another fMRI study, Trost et al. (2014) demonstrated that listening to a consonant musical beat (rated as pleasant and more arousing) resulted in faster detection of beat-synchronous targets and activation of motor-and reward-related brain areas during a visuomotor attentional task in comparison with a dissonant musical beat (rated as unpleasant and less arousing). Recently, Matthews et al. (2020) demonstrated that listening to musical rhythm rated as more pleasant and groove-inducing was associated with activation of reward-and motor-related regions, compared with musical rhythm rated as less pleasant and groove-inducing. To sum up, neuroimaging evidence substantiates the notion that rhythmic motor entrainment to music co-occurs with pleasure and reward responses. Simply stated, people want to move with music (hedonic motivation) because they like doing so (hedonic response). This evidence of affective benefits of entrainment suggests the use of synchronous music not only for performance enhancement as implied in Terry et al.’s (2020) meta-analysis but also to maximize hedonic responses and hedonic motivation for PA. Because evidence is lacking to test the specific role of entrainment in PA settings, future studies may address this by decomposing the effects of beat accentuation and/or tempo synchronization.

It should be recognized that affective and motor responses to music do not necessarily co-occur. There is music that induces pleasure but that does not necessarily generate motor reactions. For instance, it’s relaxing and pleasing to listen to Mozart’s symphonies, but such music may not be a popular choice for vigorous dance or workout sessions. The opposite case is also possible in that there are music or auditory stimuli that facilitate motor actions with little change in affective responses. An example of this case would be metronome pulses frequently used as a type of RAS for gait rehabilitation among people with PD (Lim et al., 2005; Ghai et al., 2018; Forte et al., 2021), stroke (Yoo and Kim, 2016; Ghai and Ghai, 2019), cerebral palsy (Ghai et al., 2018) and multiple sclerosis (Ghai and Ghai, 2018). None of these studies have considered participants’ hedonic responses to RAS in that their primary purpose was to stimulate the motor system. To benefit PA behaviors, however, music would be more pleasurable and motivational than isochronous pulse, as demonstrated by a study (Lim et al., 2014) in which synchronous and asynchronous music stimuli elicited greater positive affect than a synchronous metronome pulse. Gait studies also demonstrated that (1) walking with a synchronous music excerpt led to greater gait velocity and stride length than a synchronous metronome pulse (Wittwer et al., 2013) and (2) personally preferred, synchronous music evoked greater pleasure and enjoyment and more vigorous walking (greater gait velocity, stride length, and arm swing) than a synchronous, isochronous drumbeat among people with PD (Park et al., 2020). For our purpose – an affect-based approach to PA promotion – it would be effectual to use music stimulation that is personally preferred and beat-accentuated in order to maximize its effects on the affective and motor systems.

Another important construct to consider is familiarity. Personally-preferred music can induce feelings of *familiarity* based upon the extent to which a music excerpt is known to an individual (North and Hargreaves, 1998; Park et al., 2019), which can be beneficial for PA. Empirical data have shown that ratings of familiarity with a sample of music excerpts are strongly correlated with ratings of liking of the same excerpts (North and Hargreaves, 1995). In PA settings, familiarity with music has been found to beneficially influence walking behaviors (Leow et al., 2015; Park et al., 2019). This could be because familiar music facilitates rhythmic motor entrainment by having listeners more easily anticipate the timing of the beats of music to which motor events are synchronized (Damm et al., 2020; Matthews et al., 2020). A systematic review of 23 neuroimaging studies revealed that motor-related regions in the brain are the top clusters of neural activation when listening to familiar music (Freitas et al., 2018). In an acute gait trial among older adults with PD, walking in time with the salient beats of self-chosen familiar music stimulation immediately resulted in greater gait velocity, stride length, arm swing range of motion, and perceived enjoyment compared with walking with metronome pulses (Park et al., 2020) and enhancing familiarity *via* repeated listening to unfamiliar music led to increased gait parameters and enjoyment ratings (Park et al., 2021). Follow-up analyses further revealed that changes in gait parameters were associated with the degrees of perceived enjoyment, familiarity, and beat salience (Park et al., 2020; Park, 2022). Enhancing personalization and familiarity of music stimulation could bolster affective responses and ACMS for PA.

### 2.4. Sensory distraction from physical exertion

Physical exertion during PA serves as an *afferent* sensory stimulus, which delivers neuronal signals from the body to the brain through the somatosensory pathways. Some examples of this somatosensory feedback are increased HR, sweating, and muscle fatigue. In the brain, the strength of the somatosensory stimulus is perceived and can be expressed as RPE (Rejeski, 1985). The sensory stimulus from external sources (e.g., music) can distract the perception of sensory signals from the internal system (i.e., body; Damm et al., 2020). Indeed, shifting attention away from unpleasant stimuli by listening to music is a well-known non-pharmacological intervention for pain management (i.e., audio-analgesia; Kühlmann et al., 2018; Lin et al., 2020; Yu et al., 2020). Therefore, music reduces feelings of physical exertion and fatigue by redirecting the nerve impulses from somatosensory pathways to auditory pathways (Bigliassi et al., 2017). In an EEG study, Bigliassi et al. (2016) demonstrated that music listening improved the performance of an isometric maximal ankle-dorsiflexion task while decreasing theta waves (4–7 Hz) in the frontal, central, and parietal regions of the brain, which is indicative of reduction in fatigue (Craig et al., 2012). The research team further demonstrated that music listening, during an acute bout of light-to-moderate cycle ergometry, led to the reallocation of attentional focus toward external stimuli, increased the use of dissociative thoughts, and reduced neural connectivity across sensorimotor cortices in the frontal and central regions of the brain, which represent exercise thoughts and feelings (Bigliassi et al., 2017). A meta-analytic review summarized evidence from 54 studies and confirmed that music listening prior to or during PA lead to lower perceived exertion compared to PA without music (Terry et al., 2020). These findings indicate that music stimulation for PA can inhibit the feelings of discomfort and fatigue coming from physical exertion, which can help make people less likely to dislike a PA session.

Sensory-distractive effects of music co-occur with feelings of pleasure. This view was substantiated by a study in which music listening evoked positive affect in addition to attentional switching from associative to dissociative thoughts during an ankle-dorsiflexion task (Bigliassi et al., 2016). It was hypothesized that the effect of music on RPE and affect can be hindered during high-intensity PA because of the strong somatosensory signals of physical discomfort (Karageorghis et al., 2009). However, in their meta-analysis, Terry et al. (2020) demonstrated that the beneficial effects of music on RPE and affective valence were not moderated by PA intensity, implying that reduced exertion and increased positive affect can be gained by music stimulation across different intensities of PA. Evidence indicates that perceived exertion is inversely associated with positive affect among low-active people (Hardy and Rejeski, 1989; Williams et al., 2008). By simultaneously reducing RPE and enhancing pleasure, music stimulation can make a PA session more enjoyable and less painful.

## 3. Music-based interventions to promote long-term physical activity

Empirical data is lacking to support or refute the effects of music on long-term PA. A systematic review (Clark et al., 2012) identified a few trials comparing participants’ attendance rate in music-based vs. non-music-based exercise interventions in people with chronic obstructive pulmonary disease (COPD; Bauldoff et al., 2002, 2005) and dementia (Mathews et al., 2001). Although the benefits of music were implicated in these interventions, no arrangement was made for the tempo or rhythm of music to influence PA and significant differences in long-term PA were not found. A recent systematic review (Chair et al., 2021) identified 3 RCTs that examined the effects of music-based exercise interventions on long-term PA in patients with coronary heart disease (Ståhle et al., 1999; Alter et al., 2015; Clark et al., 2017). Although Ståhle et al. (1999) reported increases in self-reported PA levels 12-months after a music-based group exercise intervention compared with usual care controls in older adults after an acute coronary syndrome, the causal effects of music on PA adherence was not addressed because non-music exercise was not used as the usual care intervention.

The RCT by Alter et al. (2015) provides critical evidence that music can benefit long-term PA. Alter et al. randomly assigned midlife-to-older adults to one of three walking-for-exercise groups for a 12-week home-based cardiac rehabilitation program: (1) walking with personalized, synchronous music playlists with accentuated beats (playlists with RAS); (2) walking with personalized, synchronous music playlists without accentuated beats (playlists only); and (3) walking without music (controls). Results showed that playlists with RAS led to nearly twofold increases in accelerometer-measured weekly volumes of PA and caloric expenditure during the intervention period, compared to the other two groups. Interestingly, the higher average of weekly PA was observed at all intensities (light, moderate, and vigorous) and maintained over 12 weeks, and the weekly volume of PA was closely associated with the weekly play-counts of the playlists (*r* = 0.61). The methodology used for RAS, especially beat accentuation, may have played the key role in the treatment effects in that playlists without RAS had little effect on PA outcomes. This view would be supported by prior evidence that music with accentuated beats was found to facilitate beat perception and music-motor synchronization (Chen et al., 2006; Burger et al., 2013).

In addition to beat accentuation, temporal synchronization between music and exercise motions may also play an important role. This assertion would be upheld by minimal effects of music in other studies. In an RCT, older adults who were discharged from a cardiac rehabilitation program and got encouraged to walk-for-exercise with a personally-preferred, *tempo-asynchronous* music playlist demonstrated trivial differences in the rate of meeting PA recommendations and accelerometer-measured PA over 26 weeks compared with controls who received the same PA guidelines without music after the same rehabilitation program (Clark et al., 2017). In another RCT, patients with COPD who participated in an 8-week walking intervention with upbeat, tempo-asynchronous music playlists showed little differences in pedometer-measured total walking distance (19.1 ± 16.7 miles vs. 15.4 ± 8 miles) and self-reported volume of PA compared with patients who received the same walking intervention without music (Bauldoff et al., 2002). These findings indirectly suggest that rhythmic entrainment derived from tempo synchronization may be a key consideration in the beneficial effects of music for long-term PA.

Based on our review of empirical evidence, there are important gaps in music-based interventions in terms of their rationale and efficacy to promote PA. The methodologies employed by Alter et al. (2015) for music playlists with RAS – beat accentuation, tempo synchronization, and preference-based personalization – may play a collective role in facilitating PA and thus more studies are needed to test its efficacy. The sample – cardiac rehabilitation patients – may have also served as an important target of the music-based exercise intervention because cardiac rehabilitation is associated with high attrition rates and poor adherence to self-paced PA (Alter et al., 2015) thus allowing the intervention to appear highly efficacious because of the low starting point. Future studies can replicate this methodology to test its efficacy among the same or other populations with cardiac or neurologic conditions as well as other populations in need of PA promotion (e.g., overweight/obese, elderly, or athletes).

## 4. Putative mechanisms of music stimulation for long-term physical activity

The mechanism linking the acute effects of music to long-term PA has not been specified in the current literature. We depict this process by proposing the MASPA model as illustrated in Figure 1. This model takes into consideration the THM to further account for how music influences long-term PA. Our premise is that long-term PA is the repeated performance of acute PA on a relatively regular basis, which is often offered in the form of a behavioral intervention or exercise training. As we described above, music stimulation can induce hedonic responses during acute PA. In a long-term context, these hedonic responses serve as the inputs into the process of psychological hedonism to generate hedonic motivation, an output of psychological hedonism, directly influencing decision-making for repeating another bout of PA in the future. *Incentive conditioning* represents a “linking” process between previous hedonic responses and hedonic motivation for upcoming PA. For example, if people dislike a PA session, this negative hedonic response is linked to their reduced motivation for upcoming PA, so they are more likely to avoid another bout of PA, which decreases the chance of adopting or maintaining long-term PA. On the other hand, if they like a PA session, this positive hedonic response is linked to enhanced motivation for upcoming PA and thus they are more likely to perform another PA bout, which increases the chance of adopting or maintaining long-term PA.

We recognize that human behaviors are not entirely driven by hedonic motivation. The THM is a dual-processing model viewing behavioral outcomes as the consequence of interactions between automatic/impulsive processing and controlled/reflective processing. Williams (2018) noted that a*utomatic processing of affect* involves the learning of associations between a stimulus and affective responses (incentive conditioning). This ‘linking’ process is automatically triggered when facing a relevant stimulus or cue, consequently leading to hedonic motivation for a behavior. By contrast, *controlled processing of affect* involves anticipated affective responses to the target behavior or behavioral outcome as a function of consciously and reflectively formulated if-then expectancies. Such anticipated affective responses occur based on deliberate evaluations of previous affective responses and thus include expectations of proximal and distal affective consequences of a behavior, generating reflective motivation for a behavior. In sum, hedonic motivation occurs automatically and impulsively as a function of hedonic response and is distinguished from reflective motivation that is formulated by deliberate and controlled affective evaluation.

It should be noted that hedonic motivation and reflective motivation often work in combination for the same behavior, but sometimes contend especially in the context of healthy behaviors. According to the Affective-Reflective Theory of physical inactivity and exercise (Brand and Ekkekakis, 2018), automatic evaluation of a PA bout triggers *action impulses* that form the basis for reflective evaluation as a deliberate process generating *action plans*. Stults-Kolehmainen et al. (2020) considered action impulse as a form of ACMS for motor behaviors by defining it as “the readiness potential and/or the conscious awareness of wanting to move.” (p. 8). Ideally, action impulses and action plans need to be in unison but often they are not because, on many occasions, MVPA is automatically avoided based on negative affect. For example, going for a run typically leads to instant discomfort or displeasure coming from physical exertion whereas watching TV on a reclining sofa leads to instant comfort or pleasure by eliminating physical exertion. Hence, many people are often hedonically motivated to spend time on a couch to seek pleasure and find an excuse for skipping a run for the day to avoid displeasure (action impulse). This dread of MVPA often occurs although, for most people, MVPA is part of their exercise program (action plan) to attain its positive health benefits and a sense of accomplishment after overcoming displeasure associated with physical exertion. Therefore, enhancing affective evaluation may be an effective strategy to align action impulses with action plans to unite hedonic motivation and reflective motivation for PA.

In the acute phase of our theoretical model (marked as blue in Figure 1), we explain that music stimulation can help acute PA be associated with pleasure (or not associated with displeasure) as a function of musical pleasure and reward, rhythmic entrainment, and/or sensory distraction from physical exertion. After acute PA, the hedonic response to a PA session is more likely to be positively evaluated (or not negatively evaluated) and linked to hedonic motivation (action impulse) – in accordance with reflective motivation (action plan) – for upcoming PA, increasing the probability of engaging in PA again, possibly on a regular basis, leading to long-term PA (marked as yellow in Figure 1). The methodologies of music manipulation we suggest, beat-accentuation, tempo-synchronization, and personally-preferred music selection, can maximize the acute mechanisms of hedonic response and hedonic motivation for a PA session and consequently increase the chance of long-term behavior change. Simply stated, exercising in sync with personally-preferred, beat-accented music playlists can help people like a PA session more – or dislike it less – and thus increase the probability of regularly repeating it. Such a strategy for enhancing hedonic motivation can greatly benefit people for accomplishing PA behavior change.

This model is testable in future studies. The mediating role of musical pleasure during acute PA can be measured with scales such as the Feeling Scale (Hardy and Rejeski, 1989), the Felt Arousal Scale (Svebak and Murgatroyd, 1985), or Affective Grid (Russell et al., 1989) as well as PA enjoyment scales (Kendzierski and Decarlo, 1991; Stanley and Cumming, 2010) and their association with concurrent ACMS (e.g., wants, urges, desires, carvings, vs. aversion, dread) for PA and sedentary behaviors can be measured using the CRAVE (Cravings for Rest and Volitional Energy Expenditure) scale (Stults-Kolehmainen et al., 2021). To further test the long-term implications of the model, researchers can assess the effects of music stimulation on desire/dread for future PA and/or actual PA behaviors. Replication of the RCT by Alter et al. (2015) in other populations may greatly advance this area of science. The mechanism of rhythmic entrainment can be assessed through subjective ratings of perceived auditory-motor synchronization (Matthews et al., 2022) and/or by analyzing time series of music and motion tracking data and their association with concurrent and/or future motivation for PA and/or PA behaviors. The mechanism of sensory distraction from physical exertion has been tested by RPE (Rejeski, 1985) and its association with PA behaviors and/or PA motivation. The associations between hedonic responses, hedonic motivation, and long-term PA can be assessed using ecological momentary assessment (EMA). EMA involves repeated sampling of individuals’ real-time behaviors and experiences in natural environments, minimizing recall bias and maximizing ecological validity, and allowing for an examination of microprocesses of human behaviors in real-world contexts (Shiffman et al., 2008).

Moreover, the music methodologies we propose, beat accentuation, tempo synchronization, and personalization of music preference, can also be manipulated to test their effects on hedonic responses, hedonic motivation, and acute and long-term PA. Manipulating the temporal elements of music (e.g., tempo, beat, and rhythm) can allow researchers to test the mechanism of rhythmic entrainment and its association with other mechanisms in PA settings. It is also possible to manipulate individual music preference to test the mechanisms of musical pleasure (liking) and reward (wanting) responses. By manipulating tempo and/or loudness of music, arousal responses to music can be tested. Few studies have manipulated these components of music to assess affective responses to music and ensuing motivational or behavioral outcomes in PA settings. Future studies may address this gap in the literature.

Baseline PA level, the stage of exercise behavioral change, or age could be potential moderators of the causal effects of music with RAS on long-term PA behaviors. Given that negative affect has been observed during exercise bouts mainly at an intensity above the ventilatory or lactate threshold (Ekkekakis et al., 2011) especially in overweight/obese people (Ekkekakis and Lind, 2006; Ekkekakis et al., 2010), our theoretical model is more likely to be effective in low-active/sedentary individuals, overweight/obese people, or older adults who are expected to have low adherence to PA based on more conflicts between hedonic motivation and reflective motivation due to negative affect. Future studies can test the theoretical model with varying exercise modalities. The methodologies used for music playlists with RAS can also be combined with jogging/running, high-intensity interval training, dance, or group/individual exercise programs to enhance affective responses and hedonic motivation.

It is important to note that we built this theoretical model in consideration of previously proposed conceptual models addressing the music-PA relationship (Karageorghis, 2015; Clark et al., 2016a; Terry et al., 2020). Importantly, we are not suggesting rejection of these models but reconsidering current theoretical approaches to music-based PA interventions from the acute phase primarily for performance enhancement to the long-term phase of behavioral change. For this new perspective, we here take a theoretical approach and attempt to integrate elements from disparate literatures that have not been linked to one another within the fields of public health and behavioral medicine.

## 5. Conclusion

Physical inactivity is epidemic and jeopardizes global health. We here suggest affective, motoric, and sensory engagement in music as an innovative approach to promote PA. From a psychological and neurobiological standpoint, we expounded the putative mechanisms underlying motivational benefits of music for acute and long-term PA. Past research has shown that exercising with personally preferred, beat-accentuated, tempo-synchronous music playlists remarkably improved the amounts of PA at all intensities over 12 weeks (Alter et al., 2015). This proof-of-concept evidence of long-term PA preliminarily upholds the notion that certain types of musical stimulation could be effective beyond a single session of PA possibly for behavior change. Despite the strong preliminary data, a theoretical basis elucidating the psychological mechanism underlying the effects of music stimulation on acute and long-term PA has been lacking. Hence, this theoretical discussion is important to fill the gap in the current literature and to promote the evidence-based development and implementation of music-based interventions for PA promotion. Our notion is that the beneficial effects of music stimulation on acute and long-term PA is primarily based on hedonic principles. In other words, certain types of music can serve as an affective stimulant to help people like their workout sessions and consequently promote ‘wanting’ responses for PA to be repeated on a regular basis. Our discussion may inform future music-based interventions to gain additional success for PA promotion.

## Data availability statement

The original contributions presented in the study are included in the article/supplementary material, further inquiries can be directed to the corresponding author.

## Author contributions

KSP conceived of the hypothesis with input from DW and JE. KSP drafted an early version of the manuscript. DW and JE revised and completed the writing of the manuscript. All authors contributed to the article and approved the submitted version.

## Conflict of interest

The authors declare that the research was conducted in the absence of any commercial or financial relationships that could be construed as a potential conflict of interest.

## Publisher’s note

All claims expressed in this article are solely those of the authors and do not necessarily represent those of their affiliated organizations, or those of the publisher, the editors and the reviewers. Any product that may be evaluated in this article, or claim that may be made by its manufacturer, is not guaranteed or endorsed by the publisher.
